# Post-COVID-19 Pandemic Rebound of Macrolide-Resistant *Mycoplasma pneumoniae* Infection: A Descriptive Study

**DOI:** 10.3390/antibiotics13030262

**Published:** 2024-03-15

**Authors:** Fan-Fan Xing, Kelvin Hei-Yeung Chiu, Chao-Wen Deng, Hai-Yan Ye, Lin-Lin Sun, Yong-Xian Su, Hui-Jun Cai, Simon Kam-Fai Lo, Lei Rong, Jian-Liang Chen, Vincent Chi-Chung Cheng, David Christopher Lung, Siddharth Sridhar, Jasper Fuk-Woo Chan, Ivan Fan-Ngai Hung, Kwok-Yung Yuen

**Affiliations:** 1 Department of Infectious Diseases and Microbiology, The University of Hong Kong-Shenzhen Hospital, Shenzhen 518053, China; xingff@hku-szh.org (F.-F.X.); dengcw@hku-szh.org (C.-W.D.); yehy@hku-szh.org (H.-Y.Y.); sunll@hku-szh.org (L.-L.S.); suyx@hku-szh.org (Y.-X.S.); caihj@hku-szh.org (H.-J.C.); skflo@hku-szh.org (S.K.-F.L.); sid8998@hku.hk (S.S.); jfwchan@hku.hk (J.F.-W.C.); 2 Department of Microbiology, Queen Mary Hospital, Hong Kong SAR, China; chy731@ha.org.hk (K.H.-Y.C.); vcccheng@hku.hk (V.C.-C.C.); 3 Department of Medicine, The University of Hong Kong-Shenzhen Hospital, Shenzhen 518053, China; ronglei@hku-szh.org; 4 Department of Pediatrics, The University of Hong Kong-Shenzhen Hospital, Shenzhen 518053, China; chenjl@hku-szh.org; 5 Department of Pathology, Queen Elizabeth Hospital, Hong Kong SAR, China; lungdc@ha.org.hk; 6 Department of Pathology, Hong Kong Children’s Hospital, Hong Kong SAR, China; 7 Department of Microbiology, School of Clinical Medicine, Li Ka Shing Faculty of Medicine, The University of Hong Kong, Pokfulam, Hong Kong SAR, China; 8 Department of Medicine, The University of Hong Kong, Pokfulam, Hong Kong SAR, China

**Keywords:** *Mycoplasma pneumoniae*, macrolide resistance, pandemic, post-COVID-19

## Abstract

The rebound characteristics of respiratory infections after lifting pandemic control measures were uncertain. From January to November 2023, patients presenting at a teaching hospital were tested for common respiratory viruses and *Mycoplasma pneumoniae* using a combination of antigen, nucleic acid amplification, and targeted next-generation sequencing (tNGS) tests. The number and rate of positive tests per month, clinical and microbiological characteristics were analyzed. A rapid rebound of SARS-CoV-2 was followed by a slower rebound of *M. pneumoniae,* with an interval of 5 months between their peaks. The hospitalization rate was higher, with infections caused by respiratory viruses compared to *M. pneumoniae*. Though the pediatric hospitalization rate of respiratory viruses (66.1%) was higher than that of *M. pneumoniae* (34.0%), the 4094 cases of *M. pneumoniae* within 6 months posed a huge burden on healthcare services. Multivariate analysis revealed that *M. pneumoniae*-infected adults had more fatigue, comorbidities, and higher serum C-reactive protein, whereas children had a higher incidence of other respiratory pathogens detected by tNGS or pathogen-specific PCR, fever, and were more likely to be female. A total of 85% of *M. pneumoniae*-positive specimens had mutations detected at the 23rRNA gene, with 99.7% showing A2063G mutation. Days to defervescence were longer in those not treated by effective antibiotics and those requiring a change in antibiotic treatment. A delayed but significant rebound of *M. pneumoniae* was observed after the complete relaxation of pandemic control measures. No unusual, unexplained, or unresponsive cases of respiratory infections which warrant further investigation were identified.

## 1. Introduction

Previous studies have shown that non-pharmaceutical public health interventions including social distancing and universal masking implemented during the Coronavirus disease 2019 (COVID-19) pandemic were associated with a marked reduction in the incidence of community-onset bacteremia due to encapsulated bacteria with respiratory transmission potential, including *Streptococcus pyogenes*, *Streptococcus pneumoniae*, *Haemophilus influenzae*, and *Neisseria meningitidis*. This observation was independently confirmed by a significant reduction in the number of notifications of largely droplet or airborne infections such as scarlet fever, tuberculosis, and chickenpox [1,2]. Except for SARS-CoV-2, a marked reduction in the incidence of respiratory viruses and *Mycoplasma pneumoniae* during this period was also observed [3]. With the sudden and complete relaxation of city lockdown and other non-pharmaceutical measures in the Chinese mainland, a significant rebound of all these infections was expected in early 2023 [4,5]. However, the pattern of rebound of different respiratory infectious agents and their burden to hospitalization have not been described in such a setting.

In mainland China, prior to the COVID-19 pandemic, respiratory syncytial virus was the leading cause of acute respiratory infection in children, and influenza A and human rhinovirus were the leading causes of acute respiratory infection in adults. Furthermore, *M. pneumoniae* was the second most common bacterial pathogen that led to acute respiratory infection in all patients [6]. Other studies have shown that co-infection of *M. pneumoniae* with other respiratory pathogens was 88.94%, with macrolide-resistant *M. pneumoniae* showing a higher co-infection rate than macrolide-susceptible strains [7]. Moreover, co-infection of *M. pneumoniae* with other respiratory pathogens have also been shown to have poorer clinical outcomes [8,9,10]. Inappropriate prescription of antibiotics provides selective pressure of respiratory flora, leading to the development of macrolide resistance in *Streptococcus pneumoniae*, *Staphylococcus aureus*, and *M. pneumoniae* [11]. After the first detection of macrolide-resistant *M. pneumoniae* in 2001 [12], there was an emergence of macrolide-resistant *M. pneumoniae* around the world, with resistance rates higher in Asian countries than in other parts of the world. The macrolide resistance rates in *M. pneumoniae* in mainland China range from 69% to 100%, with A2063G mutation at 23S rRNA region being the commonest mutation [13,14,15].

In this report, we present the incidence of respiratory infectious pathogens including *M. pneumoniae* in a teaching hospital in Shenzhen, a cosmopolitan city in the Pearl River delta. Notably, there was a rapid rebound of SARS-CoV-2. What was unexpected was the gradual increase in incidence of the atypical pneumonia agent, *M. pneumoniae*, over a period of 6 months before a major outbreak surge in late 2023. This surge poses a significant burden on the hospital services, especially in the pediatric age group in both outpatients and inpatients. In this context, the clinical and microbiological characteristics of patients hospitalized for *M. pneumoniae* infection were analyzed and discussed. 

## 2. Results

**Figure 1:**
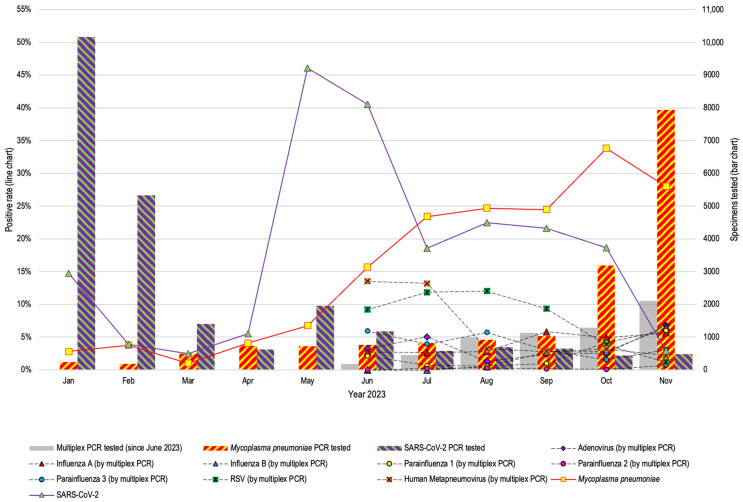
Number of respiratory specimens tested and the positive rate of different respiratory pathogens at HKU-SZH in 2023. Remarks: The respiratory multiplex PCR panel was introduced in June 2023; therefore, only data from June 2023 were available for respiratory viruses. RSV: respiratory syncytial virus.

A total of 46,718 NPS and TS specimens from 33,896 patients were tested by a combination of RT-PCR and PCR tests. The number of specimens tested and the positive rates for respiratory viruses by multiplex RT-PCR, and SARS-CoV-2 and *M. pneumoniae* monoplex PCR are illustrated in Figure 1 (Details available in Supplementary Information S1). Notably, 10,164 and 5325 specimens were tested for SARS-CoV-2 with a positive rate of only 14.8% and 3.9% in January and February of 2023, just after the opening up and relaxation of all public health measures for pandemic control. The rebound of SARS-CoV-2 appeared rapidly and peaked by May with a positive test rate of over 45%. SARS-CoV-2 testing changed from initially pre-emptive testing to on-demand testing based on fever and respiratory symptoms since 8th January 2023. While the number of requests for *M. pneumoniae* PCR tests was always over 100 per month since April, the positive rate was low at 2.8% and 3.8% in January and February, respectively, but started to increase to 6.8% by May, and then jumped to 23.4% in July, with static positive rate at 24.7% in August, and 24.5% in September, before peaking at 33.8% in October. The number of multiplex RT-PCR tests for respiratory viruses started to increase from 185 by June to 1134 by September, and the number of *M. pneumoniae* PCR tests started to increase from 499 by March to 7934 in November. In addition to SARS-CoV-2, the most common positive respiratory viruses detected were respiratory syncytial virus, influenza A virus, human adenovirus, human metapneumovirus, influenza B virus, and human parainfluenza virus types 3, 1, and 2.

Because the respiratory viruses multiplex RT-PCR panel was introduced in June 2023, only data from June to November were analyzed and compared for specimens positive for respiratory viruses and *M. pneumoniae* by pathogen-specific PCR (Table 1). There were 2694 specimens (1120 from children) positive for respiratory viruses and 4094 specimens (3687 from children) positive for *M. pneumoniae*. There were more males than females in both groups. The median age of patients with respiratory virus infection was higher than that of patients with *M. pneumoniae* infection (median age 35 vs. 7). The hospitalization rate of patients with respiratory viruses was higher than that of patients with *M. pneumoniae* infection (47.8% vs. 35.1%). A similar observation in hospitalization rates was seen in the pediatric population (66.1% vs. 34.0%, respectively).

A total of 3486 *M. pneumoniae* PCR-positive patients were identified from September to November. Over 90% were younger than 18 years old (children) with a median age of 7 years. For adults, the median age was 36 years. More males than females were found in children but the number of females was almost twice the number of males in adults (Table 2). 

A total of 1049 children and 99 adults were hospitalized during this period (Table 2). The number of tests (*M. pneumoniae* and SARS-CoV-2 monoplex PCR, respiratory viruses multiplex RT-PCR, and tNGS) received by each group were included in Supplementary Table S1. Again, there were significantly more females in adults but more males than females in children. Significantly more adults than children had underlying chronic medical illnesses (34.3% vs. 4.0%, *p* < 0.001). The most frequent underlying chronic diseases in children were hematological diseases, atopy, cardiovascular diseases, and chronic renal diseases. In adult patients, chronic pulmonary diseases, chronic liver impairment, and solid organ malignancies were more common. Fever and cough were the main presenting symptoms in both age groups, but adults more significantly had headache, sore throat, fatigue, and shortness of breath. Rash and neurological manifestations were rare. The duration of hospitalization of the 1148 patients ranged from 0 to 17 days, with a median of 4 days. There were no significant differences in the duration of hospitalization between the children and adults group.

Out of 1148 patients, 952 children (90.8%) and 83 adults (83.8%) had pulmonary infiltrates manifested as localized opacities with unclear boundaries or interstitial changes on chest X-ray or Computed Tomography of the thorax (Table 3). Concomitant consolidations were observed in 149 patients, with more adults having consolidations than children (*p* < 0.001). For blood parameters, most patients did not have leukocytosis or neutrophilia. Consistent with the more severe clinical symptoms and radiological consolidation, the serum C-reactive protein levels of adults were three times that of the children, but this phenomenon was not observed for serum procalcitonin. In multivariate analysis, symptoms of fatigue, higher serum CRP level, and underlying medical comorbidities were more significantly observed in hospitalized adult patients, while hospitalized pediatric patients were more likely to have symptoms of fever, other respiratory pathogens detected by tNGS or pathogen-specific PCR, and more likely to be females (Supplementary Table S2).

The percentage of *M. pneumoniae*-positive specimens with macrolide-resistant mutations detected by tNGS was approximately 85% (749/885). Furthermore, tNGS showed that most (747/749, 99.73%) of our macrolide-resistant *M. pneumoniae* isolates had A2063G mutation, and only two isolates had A2064G mutation. Significantly more children (over 26%) had other respiratory pathogens detected in tNGS, SARS-CoV-2 PCR, and respiratory viruses multiplex RT-PCR (Table 2 and Supplementary Figure S1). The *M. pneumoniae* bacterial load of these 811 children was significantly higher than that of 75 adults. A total of 770 patients who had both quantitative PCR and tNGS were analyzed. There was a positive correlation between the bacterial load and normalized sequence reads by tNGS (r = 0.3774, *p* < 0.001). 

**Figure 2:**
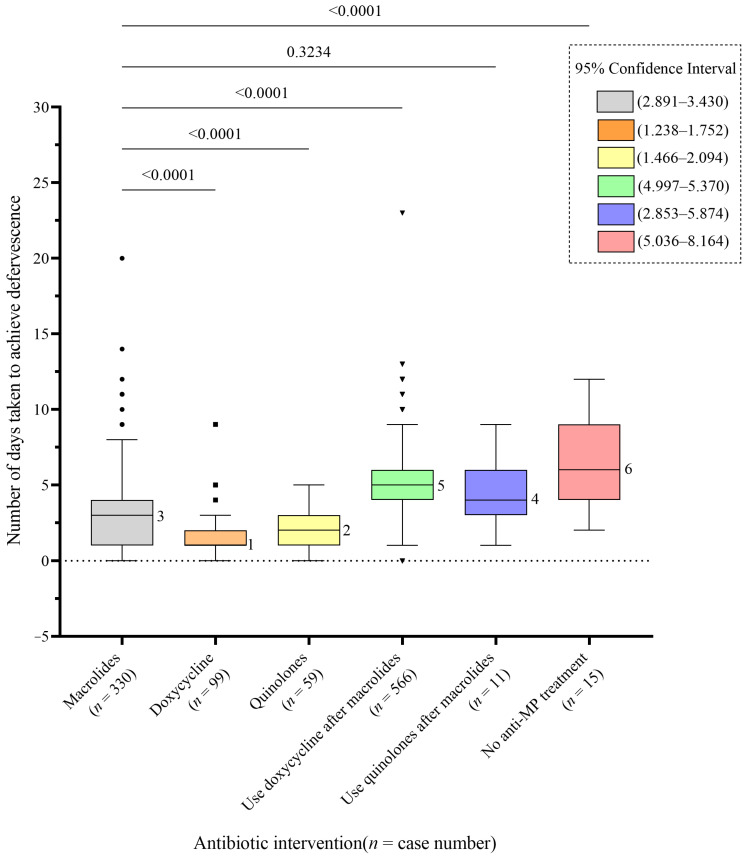
Days needed to achieve defervescence with different antimicrobial regimens for treatment of *Mycoplasma pneumoniae* infection. Remarks: A total of 1065 of the 1148 patients achieved defervescence after initiation of different antibiotics, and 15 patients did not receive any drugs with anti-*Mycoplasma* activity. A total of 9 of the 15 patients did not receive any antibiotics and the other 6 received only beta-lactam antibiotics. Each bar represents the number of days to achieve defervescence with different interventions. The middle line in the box represents the median, the edges of the box represent the interquartile range, the thin black line represents the range, and the circles, squares, and triangles on the plot represent each data point outside the interquartile range of respective groups. MP: *Mycoplasma pneumoniae*.

For the antibiotic treatment, 942 patients were initially treated with macrolides and 112 were initially treated with doxycycline. None of the patients died or required admission into ICU for respiratory support. Excluding 37 patients whose fever had resolved before antibiotic treatment, the time to defervescence was shorter in patients who started on macrolides than those who were not treated by any anti-*M. pneumoniae* antibiotics (median: 3 days vs. 6 days, *p* < 0.001). A longer defervescence time was observed in patients who required switching to active agents such as doxycycline (median: 3 days vs. 5 days, *p* < 0.001). Furthermore, the time to defervescence was significantly longer in patients who initially started on macrolides when compared to those who initially started on doxycycline (median: 3 days vs. 1 day, *p* < 0.001) or fluoroquinolones (median: 3 days vs. 2 days, *p* < 0.001) (Figure 2). 

A total of 19 patients (10 girls and 9 boys) achieved defervescence after antibiotic treatment for more than 10 days. The age of these patients ranged from 9 months to 12 years, with none having underlying diseases. A total of 16 of the patients had a macrolide resistance mutation detected, and 12 patients had other respiratory pathogens detected in tNGS, SARS-CoV-2 PCR, and respiratory viruses multiplex RT-PCR. A total of 10 patients had a delay in administration of doxycycline, while 4 did not switch to doxycycline despite infection by macrolide-resistant *M. pneumoniae*.

In three of the children with mono-infection of *M. pneumoniae* with persistent fever despite an antibiotic switch from macrolides to doxycycline, one had massive pleural effusion, and two had large consolidation and rash, respectively. One patient with macrolide-susceptible *M. pneumoniae* developed persistent low-grade fever despite a course of macrolide; therefore, another course of macrolide was prescribed to that patient, with a total duration of fever of 11 days.

## 3. Discussion

The abrupt relaxation of social distancing and universal masking in early 2023 provided a unique opportunity to study the rebound characteristics of respiratory pathogens in patients who presented to the HKU-SZH. Unlike other respiratory viruses, the time to rebound of *M. pneumoniae* appeared to be longer and posed a significantly greater burden on the hospital services after the relaxation of the city lockdown and nonpharmaceutical measures. The interval between the peaks in the rebound of SARS-CoV-2 (May 2023) and *M. pneumoniae* (October 2023) was 5 months. Comparison of cases of respiratory viruses and *M. pneumoniae* infections indicated that respiratory viruses more severely affected older individuals than *M. pneumoniae*. The high hospitalization rate in adults with respiratory virus infections could be attributed to the exacerbating effects of underlying chronic illnesses such as a chronic pulmonary disease. In the pediatric population, the hospitalization rate was higher in children with respiratory virus infections. However, the absolute number of children admitted due to *M. pneumoniae* was almost 1.7-fold higher (1253/740, Table 1), highlighting the significant burden on the hospital services during this period of time.

Unlike influenza viruses which tend to affect the elderly more severely than children, *M. pneumoniae* significantly affected school children (median age of 7) and younger adults (median age 36). Among adults, *M. pneumoniae* affected more females, and the infection presented with more severe clinical, radiological, and biochemical manifestations, as indicated by the levels of C-reactive protein. On the other hand, children had a higher incidence of other respiratory pathogens detected by tNGS, SARS-CoV-2 PCR, and respiratory viruses multiplex RT-PCR. 

In our study, tNGS was used for measurement of bacterial load and detection of mutations associated with macrolide resistance. Although there was a positive correlation between the bacterial load detected by tNGS and quantitative PCR, the low but significant correlation coefficient can be attributed to differences in the timing of specimen collection for various tests. A high incidence (approximately 85%) of macrolide resistance was also found in our cohort. Patients treated with doxycycline and fluoroquinolones improved more rapidly in terms of fever duration when compared with those treated with macrolides. When compared with those not treated or treated by ineffective antibiotics such as beta-lactams, patients with macrolide-resistant *M. pneumoniae* infection treated by macrolides had a short time to defervescence. This observation is not surprising because macrolides are known to have immunomodulatory functions [16] and might dampen down the proinflammatory cytokines induced by *M. pneumoniae* before the host immune response controls bacterial replication [17]. However, treating an infection caused by macrolide-resistant bacteria with macrolides may suppress the macrolide sensitive normal flora which may provide a better competitive advantage for the mycoplasma infection and even prolong the bacterial shedding and thus the outbreak.

The prolonged interval for the rebound of *M. pneumoniae* infection after relaxation of the pandemic control measures could be attributed to several factors. Firstly, *M. pneumoniae* has a slow growth rate, with a generation time of 6 h. Additionally, it has a long incubation period of 2–3 weeks. Transmission of the infection requires close contact between individuals [18]. These factors may have contributed to the delayed resurgence of the infection. Secondly, the absence of community circulation of *M. pneumoniae* during three years of the SARS-CoV-2 pandemic strict lockdown probably led to the accumulation of a large number of highly susceptible children who have not previously been exposed to *M. pneumoniae*, as a consequence of school closures. The persistent shedding of this bacteria for over 7 weeks and a large number of asymptomatic carriers, especially in family members and school contacts, would finally have led to a high attack rate within the susceptible population [18,19]. Thirdly, the most commonly used antibiotics for respiratory tract infections are macrolides such as azithromycin, or beta-lactams such as amoxicillin-clavulanate, in both children and adults at the outpatient and inpatient settings. It can be inferred that patients with the common typical pneumonic agents such as *Streptococcus pneumoniae*, *Haemophilus influenzae*, and *Staphylococcus aureus* would have been well treated by either group of antibiotics, while patients with the macrolide-resistant atypical pneumonic agent such as *M. pneumoniae* would not improve with this management strategy. A proper review of the treatment protocol should be undertaken since doxycycline is the drug of choice for macrolide-resistant *M. pneumoniae* and a short course of doxycycline should not cause dental and bone complications even in children younger than 8 years of age [20,21].

In terms of gender differences in disease susceptibility, the mice challenge experiment conducted with *M. pulmonis* demonstrated that male mice had more severe lung parenchymal inflammatory damage than female mice [22]. The finding suggests that there is a possibility of gender differences in terms of the severity of *Mycoplasma* infection, therefore explaining our study finding of more male cases observed in the children group, similar to another study performed during the COVID-19 pandemic [23]. However, the finding of more female cases observed in the adult group was unexpected. Although gender differences in *M. pneumoniae* infection in adults is not consistently observed in the literature, higher seroprevalence in females was previously reported in Taiwan [24]. Further research is required to understand whether this gender difference could be explained by social factors, such as housewives spending more time with children, thereby increasing the risk of acquiring *M. pneumoniae*.

There are several limitations in this study. First, this observational study was performed in a single hospital in the Chinese mainland, therefore limiting the generalizability of the findings. Furthermore, there was no standardized protocol for requesting the type of viral and mycoplasma assays. The testing decisions were made by the attending clinician on an individual patient basis. Therefore, there is a possibility that patients with co-infection with respiratory viruses and *M. pneumoniae* were not picked up in this study. Moreover, the lack of significant differences between the adult and children groups could be due to the limited number of adult patients. However, the small number of adult patients in this study was an expected result, as *M. pneumoniae* infection predominantly affects children. In addition, although our study showed that the bacterial load of *M. pneumoniae* was higher in the pediatric population, the difference could be related to the quality of specimen collection. Furthermore, due to the retrospective nature of this study, the causal relationship between the treatment of *M. pneumoniae* infection with clinical improvement is difficult to establish. However, the findings of our study were consistent with previous literature that the use of appropriate treatment with doxycycline or fluoroquinolones allows more rapid improvement clinically when compared with macrolides in the treatment of macrolide-resistant *M. pneumoniae* [25]. With the high resistance rate observed in mainland China [13,14,15], there is a need for a change in the treatment protocol for community-acquired pneumonia, especially in children. 

## 4. Materials and Methods

Between January and November 2023, all outpatients and inpatients presenting to The University of Hong Kong-Shenzhen Hospital (HKU-SZH) with unexplained fever or respiratory symptoms including cough, sputum, shortness of breath, chest pain, sore throat, or rhinorrhea were sampled by nasopharyngeal swab (NPS), throat swab (TS), or bronchoalveolar lavage (BAL). Different tests for respiratory pathogens were available in our laboratory including the influenza A and B viruses antigen test, real-time qualitative 8-multiplex RT-PCR for respiratory viruses which included the 6-multiplex RT-PCR for human adenovirus, human metapneumovirus, respiratory syncytial virus, and human parainfluenza virus types 1, 2, 3, and 2-multiplex RT-PCR for influenza A and B viruses, SARS-CoV-2 RT-PCR, and real-time quantitative *M. pneumoniae* PCR. The testing decision was made by the attending clinician on an individual patient basis. For hospitalized patients with *M. pneumoniae* infection, especially children who were suspected to have macrolide-resistant *M. pneumoniae* infection and/or co-infection with other pathogens, the sample would also be tested by targeted next-generation sequencing (tNGS) for 198 known human pathogens and their antimicrobial resistance genes. The clinical data of the hospitalized patients and microbiological data were collected from the hospital clinical information system and the hospital laboratory information system, respectively. Macrolide-resistant *M. pneumoniae* was defined as detection of known resistance mutation (A2063G, A2064G, A2067G, and C2617G) at the 23S rRNA region of *M. pneumoniae*.

Descriptive statistics were reported as median with interquartile range (IQR). Frequency with percentages were reported for categorical variables. Student’s *t*-test or the Mann–Whitney U test was used for two-group comparisons of continuous variables depending on the distribution of the variable. The Chi-square test was performed for two-group comparisons for categorical variables. For comparison between hospitalized *M. pneumoniae* adult and pediatric patients, variables that were considered as statistically significant in univariate analysis were subjected to multivariable analysis by binomial logistic regression using backward stepwise regression. Bacterial load by quantitative PCR was excluded from the multivariable analysis as many factors may affect the bacterial load such as specimen quality. Statistical analyses were performed using SPSS version 26.0 and GraphPad Prism version 6.0. A *p*-value less than 0.05 was considered statistically significant.

### 4.1. The Influenza A and B Antigen Test

Briefly, the influenza virus type A and type B Antigen test (ClearView) is a lateral-flow immunoassay that detects both influenza A and B viruses. The test was performed with fresh clinical specimens according to the manufacturer’s instructions (Abbott, Shanghai, China) [26]. The detection limit of this kit is 1.25 × 10^3^ Chicken Embryo Lethal Dose 50 (CELD_50_) for Influenza A (A2/Aichi/2/68 H3N2) and 0.50 × 10^2^ CELD_50_ for influenza B (Hong Kong/5/72). 

### 4.2. Real-Time Quantitative Mycoplasma Pneumoniae PCR

A volume of 10 µL DNA was concentrated-lysed extracted from the TS and real-time quantitatively amplified for the conserved region of the *M. pneumoniae* genome (Mp181) with internal control RNaseP according to the manufacturer’s protocol (Sansure Biotech Inc., Hunan, China) [27]. The detection limit of this kit is 4.00 × 10^2^ copies/mL. The linear detection sensitivities ranged from 4.00 × 10^2^ to 4.00 × 10^7^ *M. pneumoniae* copies/mL of sample.

### 4.3. Real-Time Qualitative SARS-CoV-2 PCR

A volume of 5 µL RNA was extracted from a 300 µL TS sample transported in 3 mL guanidine hydrochloride transport medium and real-time qualitatively amplified for the conserved regions ORF1ab and N genes of the SARS-CoV-2 with internal control RNaseP according to the manufacturer’s protocol (Bio-Germ Biotech Inc., Shanghai, China) [28]. The detection limit of this kit is 1.50 × 10^2^ copies/mL.

### 4.4. Real-Time Qualitative 8-Multiplex Respiratory Virus RT-PCR Assay: 6-Multiplex Non-Influenza Respiratory Viruses and 2-Multiplex Influenza A and Influenza B RT-PCR Kits

For the 6-multiplex Respiratory Viral RT-PCR test in 2 reaction tubes, 5 µL RNA/DNA was extracted from a 300 µL TS sample transported in 3 mL guanidine hydrochloride transport medium and RT-PCR with real-time amplification for the conserved gene regions of human adenovirus, human metapneumovirus, and human parainfluenza virus type 1, and internal control RNaseP in one tube, and for respiratory syncytial virus, human parainfluenza virus types 2, 3, and internal control RNase P in another tube, according to the manufacturer’s protocol (Bio-Germ Biotech Inc., Shanghai, China) [29]. The detection limit of this kit is 1.00 × 10^3^ copies/mL.

For 2-multiplex influenza virus type A/B nucleic acid test, RT-PCR was performed using the same procedure and temperature profiles as for the 6-multiplex assay as above, 5 µL RNA was amplified for the conserved regions of M protein gene of Influenza A virus, N protein gene of influenza B virus, and internal control RNaseP in a tube according to the manufacturer’s protocol (Bio-Germ Biotech Inc., Shanghai, China) [30]. The detection limit of this kit is 1.00 × 10^3^ copies/mL.

### 4.5. Next-Generation Sequencing

The TS, sputum, and BAL samples were sent to a commercial company laboratory for targeted next-generation sequencing (tNGS) analysis for respiratory tract pathogens and drug-resistance genes (Respiratory 100 panel, KingMed Diagnostics, Guangzhou, China). It achieves an early diagnosis of respiratory infection by detecting 198 pathogens including 80 bacteria, 79 viruses (35 DNA viruses and 44 RNA viruses), 32 fungi, 7 mycoplasmas/chlamydia, and 3 kinds of antimicrobial-resistant organisms at 15 genes (carbapenem-resistant Enterobacterales, methicillin-resistant *Staphylococcus aureus*, and macrolide-resistant *M. pneumoniae* at 4 mutation points of 23S rRNA at A2063G, A2064G, A2067G, and C2617G) according to the manufacturer’s information (Details available in Supplementary Information S2). Following nucleic acid extraction, specific primers are designed to target-specific bacterial, mycobacterial, viral, and fungal sequences, as well as some selected antimicrobial resistance sequences for enrichment of multiplex targets, followed by library preparation for NGS sequencing [31,32]. The reads of sequence (normalized sequence number in 100,000 primary sequences detected) shown in the report were calculated based on internal control. When estimating the concentration of the target pathogen, exogenous plasmids of known concentrations were used as the internal control. The number of normalized sequence reads of the target pathogen could be calculated using the amplification efficiency ratio of the internal control.

## 5. Conclusions

In conclusion, following the emergence of SARS-CoV-2 from the Chinese mainland, there was international concern when mainland hospitals in the northern part of China were reported to have been overwhelmed by attendees with febrile respiratory illnesses in late 2023. However, in Southern China, we did not observe any unusual, unexplained, or unresponsive cases of respiratory infection which required further investigation by unbiased metagenomic next-generation sequencing. This significant winter outbreak of *M. pneumoniae* infections which occurred 5 months after the surge of respiratory virus infections can be attributed to an immunity gap and therefore a high population susceptibility resulting from the lack of exposure to these agents during the last three pandemic years. The high rate of macrolide resistance in *M. pneumoniae* re-emphasized the need to review treatment protocols for acute respiratory infections in mainland China.

## Figures and Tables

**Table 1:** Characteristics of patients with specimens positive for respiratory viruses and *Mycoplasma pneumoniae* by pathogen-specific PCR from June to November 2023.

	Respiratory Viruses	*M. pneumoniae*
Total No. of +ve specimens	2694 ^1^	4094
No. of +ve specimens in children	1120	3687
Female/Male	1264/1430	1961/2133
Age (years), median (IQR)	35 (4, 65)	7 (5, 9)
Hospitalization (%)	1289 (47.8%)	1438 (35.1%)
Hospitalization in children (%)	740 (66.1%)	1253 (34.0%)

^1^ More than one respiratory virus was detected in 70 specimens. A total of 297 patients have more than one respiratory specimen sent.

**Table 2:** Clinical characteristics of all patients or hospitalized patients with *Mycoplasma pneumoniae* infection from September to November 2023.

	Children (Younger Than 18 Years)	Adults	*p*-Value
All *Mycoplasma pneumoniae* infection			
No. of specimens	3216 (90.5%)	339 (9.5%)	
Female/Male	1462/1754	231/108	<0.001
Median age (IQR)	7 years (5, 9)	36 years (32, 41)	
Hospitalized *Mycoplasma pneumoniae* infection			
No. of patients	1049	99	
Female/Male	448/561	72/27	<0.001
Median age (IQR)	6 years (5, 8)	35 years (31, 41)	
No. of patients with underlying disease(s)	42 (4.0%)	34 (34.3%)	<0.001
Underlying chronic diseases (>1 disease in some patients)			
Atopy (rhinitis, asthma, dermatitis)	6	2	
Malnutrition	2	0	
Thyroid nodule	1	3	
Hypertension	0	3	
Diabetes mellitus	0	4	
Hyperuricemia	1	4	
Cardiovascular disease	4	3	
Pulmonary disease	0	8	
Chronic renal impairment	4	3	
Chronic liver impairment	0	8	
Gastrointestinal disease	2	2	
Neurological disease	3	3	
Solid organ malignancies	0	6	
Hematological diseases	17	5	
Precocious puberty	1	0	
Down syndrome	1	0	
Symptoms and signs			
Fever	1036 (98.8%)	92 (92.9%)	<0.001
Cough	1043 (99.4%)	99 (100%)	1.000
Fatigue	10 (1.0%)	10 (10.1%)	<0.001
Shortness of breath	29 (2.8%)	7 (7.1%)	0.030
Headache	13 (1.2%)	12 (12.1%)	<0.001
Sore throat	34 (3.2%)	12 (12.1%)	<0.001
Asthma	12 (1.1%)	2 (2.0%)	0.344
Rash	27 (2.6%)	0 (0.0%)	0.085
Neurological manifestations	8 (0.8%)	3 (3.0%)	0.062
Median days of hospitalization (IQR)	4 (3–6)	4 (3–5)	0.224
Favorable outcome ^1^	1046 (99.7%)	99 (100%)	1.000

^1^ Three children were discharged without defervescence.

**Table 3:** Investigation findings of hospitalized patients with *Mycoplasma pneumoniae* infection from September to November 2023.

	Children (Less Than 18 Years)	Adults	*p*-Value
Radiology			
Pulmonary infiltrates	952 (90.8%)	83 (83.8%)	0.027
Consolidation	117 (11.2%)	32 (32.3%)	<0.001
Pleural effusion	40 (4.4%)	6 (6.1%)	0.273
Lab test, median (IQR)			
White blood cell count (×10^9^/L)	7.10 (5.73, 8.94)	7.08 (5.87, 8.49)	NA ^1^
Neutrophil count (×10^9^/L)	4.21 (3.17, 5.64)	4.93 (4.04, 6.31)	NA ^1^
Lymphocyte count (×10^9^/L)	1.99 (1.51, 2.58)	1.44 (1.10, 1.76)	NA ^1^
Monocyte count (×10^9^/L)	0.59 (0.45, 0.78)	0.61 (0.44, 0.79)	NA ^1^
Platelet count (×10^9^/L)	263 (219, 321)	225 (190, 279)	NA ^1^
Hemoglobin (g/L)	125 (119, 131)	129 (122, 139)	NA ^1^
Alanine aminotransferase (U/L)	12.6 (10.4, 16.33)	17.75 (12.33, 28.70)	NA ^1^
Aspartate aminotransferase (U/L)	28 (23.8, 33.5)	19.3 (16.2, 25.0)	NA ^1^
Total bilirubin (μmol/L)	4.6 (3.5, 6.0)	8.1 (5.4, 10.8)	NA ^1^
Direct bilirubin (μmol/L)	2.0 (1.5, 2.5)	3.5 (2.6, 4.4)	NA ^1^
Albumin (g/L)	41.8 (40.2, 43.4)	41.6 (38.8, 43.1)	NA ^1^
Creatinine (μmol/L)	40 (34, 47)	63 (54, 72)	NA ^1^
C-reactive protein (mg/L)	11 (5.1, 20)	35.34 (15.09, 70.70)	<0.001
Procalcitonin (ng/mL)	0.15 (0.10, 0.24)	0.09 (0.06, 0.13)	NA ^1^
*M. pneumoniae* DNA by quantitative PCR (copies/mL)	102,000 in 811 patients (124,500, 533,500)	25,000 in 75 patients (7300, 184,000)	0.002
Normalized sequence reads of *M. pneumoniae* detected via tNGS (per 100 kb primary sequence reads)	69,045 in 870 patients (1484, 21,983.75)	41,144 in 10 patients (7323.75, 46,504.25)	NA ^2^
Detection of other respiratory pathogens in tNGS and pathogen-specific PCR	275 (26.2%)	2 (2.0%)	<0.001

^1^ NA: not applicable, as the findings are generally within the normal range, and the difference can be explained by the normal physiological differences between age group; ^2^ NA: not applicable due to the low number of adult specimens tested.

## Data Availability

The data that support the findings of this study are available from the corresponding author, K.Y. Yuen, upon reasonable request.

## Supplementary Materials

The following supporting information can be downloaded at: https://www.mdpi.com/article/10.3390/antibiotics13030262/s1. Supplementary Information S1. Number of specimens and positive specimens for respiratory pathogens tested in HKU-SZH hospital. Supplementary Information S2. Pathogens that can be detected by the target next-generation sequencing in this study. Supplementary Table S1. The number of tests received by hospitalized pediatric and adult patients with *M. pneumoniae* infection from September to November 2023. Supplementary Table S2. Multivariate analysis on factors associated with hospitalized adult patients infected with *M. pneumoniae*. Supplementary Figure S1. Number of patients with other respiratory pathogens as identified by tNGS and/or pathogen-specific PCR assay in the 277 hospitalized patients with *M. pneumoniae* infection in this study.
