# Supplementary material for: Post-COVID-19 Pandemic Rebound of Macrolide-Resistant Mycoplasma pneumoniae Infection: A Descriptive Study
Source: Antibiotics (Basel). 2024 Mar 15;13(3):262. doi: 10.3390/antibiotics13030262 (PMC10967482; doi:10.3390/antibiotics13030262)
Supplement: Supplementary file 1 [file antibiotics-13-00262-s001.zip › Supplementary Figure S1.pdf]

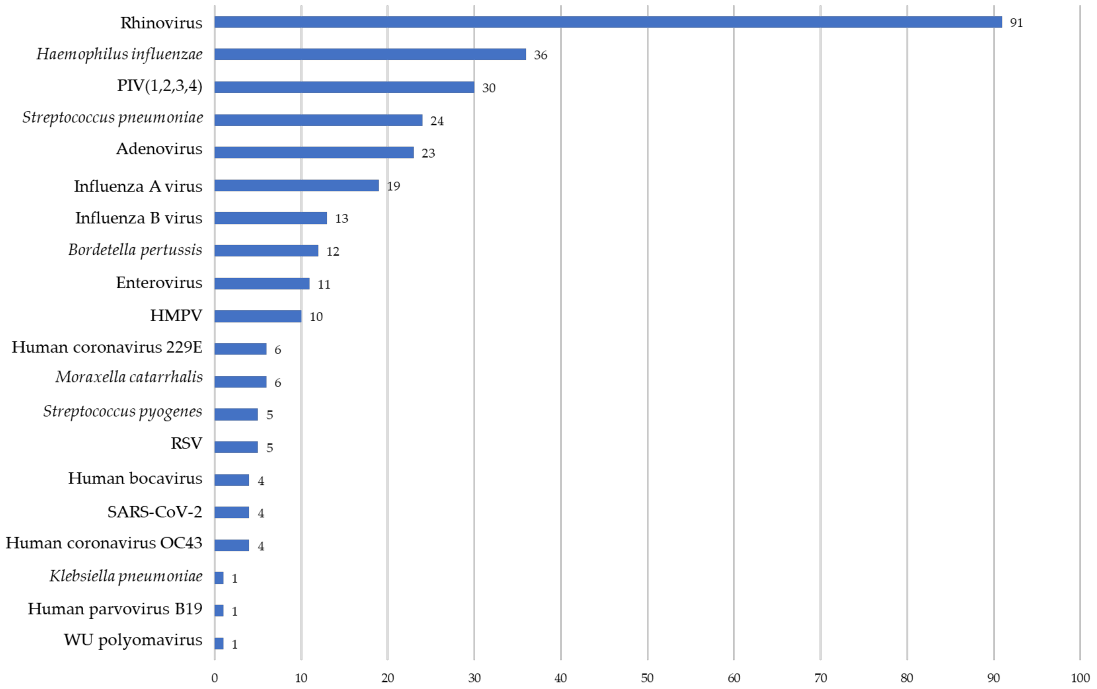

Supplementary Figure S1. Number of patients with other respiratory pathogens as identified by tNGS and/or pathogen-specific PCR assay in the 277 hospitalized patients with *M. pneumoniae* infection in this study. Remarks: WU polyomavirus has doubtful pathogenic significance in respiratory infection. PIV: parainfluenza virus; RSV: respiratory syncytial virus; SARS-CoV-2: severe acute respiratory syndrome coronavirus 2; HMPV: human metapneumovirus.
