# Supplementary material for: Post-COVID-19 Pandemic Rebound of Macrolide-Resistant Mycoplasma pneumoniae Infection: A Descriptive Study
Source: Antibiotics (Basel). 2024 Mar 15;13(3):262. doi: 10.3390/antibiotics13030262 (PMC10967482; doi:10.3390/antibiotics13030262)
Supplement: Supplementary file 1 [file antibiotics-13-00262-s001.zip › Supplementary Information S2.pdf]

Supplementary Information S2. Pathogens that can be detected by the target next-generation sequencing in this study.

The TS, sputum, and bronchoalveolar lavage (BAL) samples were sent to a commercial company for targeted next-generation sequencing (tNGS) analysis for respiratory tract pathogens and drug-resistance genes (Respiratory 100 panel, KingMed Diagnostics, China). It achieves an early diagnosis of respiratory infection by detecting 198 pathogens including 80 bacteria (42 Gram-positive bacteria including *Corynebacterium diphtheriae*, *Mycobacterium* species, *Nocardia* species, *Parvimonas micra*, *Rhodococcus* species, *Staphylococcus aureus*, *Streptococcus* species, *Tropheryma whipplei*, and *Trueperella pyogenes*; 38 Gram-negative bacteria including *Acinetobacter* species, *Bacteroides fragilis*, *Bordetella* species, *Brucella* species, *Burkholderia* species, *Elizabethkingia* species, *Enterobacter cloacae* complex, *Escherichia coli*, *Fusobacterium* species, *Haemophilus influenzae*, *Klebsiella* species, *Legionella* species, *Moraxella catarrhalis*, *Neisseria meningitidis*, *Pasteurella multocida*, *Proteus mirabilis*, *Pseudomonas aeruginosa*, *Serratia marcescens*, and *Stenotrophomonas maltophilia*), 79 viruses (35 DNA viruses including BK polyomavirus, JC polyomavirus, WU polyomavirus, Herpesviridae, Human bocavirus, Human mastadenovirus, and Human parvovirus B19; and 44 RNA viruses including Coxsackievirus, Echovirus, Enterovirus, Human coronavirus 229E, HKU1, NL63, OC43, Human metapneumovirus, Human orthobulavirus, Human respirovirus, Human respiratory syncytial virus, Influenza A, B, C, Measles morbillivirus, Mumps orthorubulavirus, Rhinovirus, Rubella virus, and SARS-CoV-2), 32 fungi (including *Aspergillus* species, *Candida* species, *Cryptococcus* species, *Fusarium* species, *Histoplasma capsulatum*, *Lichtheimia* species, *Lomentospora prolificans*, *Meyerozyma guilliermondii*, *Mucor* species, *Nakaseomyces glabrata*, *Pichia kudriavzevii*, *Pneumocystis jirovecii*, *Rhizomucor* species, *Rhizopus* species, *Scedosporium* species, *Talaromyces marneffei*, and *Trichosporon asahii*), 7 mycoplasmas/chlamydia including *Chlamydia pneumoniae*, *Chlamydia psittaci*, *Chlamydia trachomatis*, *Coxiella burnetii*, *Mycoplasma pneumoniae*, *Ureaplasma parvum*, and *Ureaplasma urealyticum*, and 3 kinds of antimicrobial-resistant organisms at 15 genes (carbapenem-resistant Enterobacterales, methicillin-resistant *Staphylococcus aureus*, and macrolide-resistant *M. pneumoniae* at 4 mutation points of 23S rRNA at A2063G, A2064G, A2067G, and C2617G) according to the manufacturer's information.
