# Supplementary material for: Post-COVID-19 Pandemic Rebound of Macrolide-Resistant Mycoplasma pneumoniae Infection: A Descriptive Study
Source: Antibiotics (Basel). 2024 Mar 15;13(3):262. doi: 10.3390/antibiotics13030262 (PMC10967482; doi:10.3390/antibiotics13030262)
Supplement: Supplementary file 1 [file antibiotics-13-00262-s001.zip › Supplementary Tables S1 and S2.pdf]

Supplementary Table S1. The number of tests received by hospitalized pediatric and adult patients with *M. pneumoniae* infection from September to November 2023.

Number of patients	Children	Adult
***M. pneumoniae* PCR only**	454	6
***M. pneumoniae* PCR with SARS-CoV-2 PCR only**	1	3
***M. pneumoniae* PCR with respiratory viruses multiplex RT-PCR only**	562	44
***M. pneumoniae* PCR, SARS-CoV-2 PCR, and respiratory viruses multiplex RT-PCR**	32	46
**Total number of tNGS performed**	870	10
**Total number of patients**	1049	99

Supplementary Table S2. Multivariate analysis on factors associated with hospitalized adult patients infected with *M. pneumoniae*.

	Odds ratio	95% Confidence Interval	p-value
**Female**	0.544	0.304 – 0.974	0.040
**Underlying medical condition**	14.114	6.937 – 28.715	< 0.001
**Fever**	0.116	0.028 – 0.475	0.003
**Fatigue**	8.723	2.212 – 34.395	0.002
**Shortness of breath**	-	-	0.119
**Headache**	-	-	0.052
**Sore throat**	-	-	0.802
**Chest X-ray showing pulmonary infiltrates**	-	-	0.054
**CRP level**	1.047	1.036 – 1.058	< 0.001
**Other respiratory pathogens detected by tNGS or pathogen-specific PCR**	0.048	0.011 – 0.221	< 0.001

Remarks: Odds ratio > 1 suggests that factor is more associated with adult patients.
